# Analysing Parallel Strategies to Alter the Host Specificity of Bacteriophage T7

**DOI:** 10.3390/biology10060556

**Published:** 2021-06-20

**Authors:** Ákos Avramucz, Christian Møller-Olsen, Aurelija M. Grigonyte, Yanahan Paramalingam, Andrew Millard, Antonia P. Sagona, Tamás Fehér

**Affiliations:** 1 Synthetic and Systems Biology Unit, Biological Research Centre, Eötvös Loránd Research Network (ELKH), 6726 Szeged, Hungary; avramucz.akos@brc.hu; 2 Doctoral School in Biology, University of Szeged, 6726 Szeged, Hungary; 3 School of Life Sciences, University of Warwick, Coventry CV4 7AL, UK; C.Moller-Olsen@warwick.ac.uk (C.M.-O.); grigonyt@ualberta.ca (A.M.G.); yanahan.paramalingam.1@warwick.ac.uk (Y.P.); 4 Department Genetics and Genome Biology, University of Leicester, Leicester LE1 7RH, UK; adm39@leicester.ac.uk; 5 Warwick Integrative Synthetic Biology Centre, University of Warwick, Coventry CV4 7AL, UK

**Keywords:** bacteriophage, phage, host specificity, host range, RBP, tail fibres, BRED

## Abstract

**Simple Summary:**

The problem of antimicrobial resistance is prominent and new alternatives to antibiotics are necessary. Bacteriophages are viruses that target host bacteria and can be used efficiently for their antibacterial properties to solve the problem of antimicrobial resistance. In this study, we explore ways to genetically modify T7 bacteriophage and make its tropism broader, so that it can attack a higher variety of bacteria. We are using different methodologies to achieve this, among of those bacteriophage recombineering using electroporated DNA (BRED), which seems to be the most efficient.

**Abstract:**

The recognition and binding of host bacteria by bacteriophages is most often enabled by a highly specific receptor–ligand type of interaction, with the receptor-binding proteins (RBPs) of phages being the primary determinants of host specificity. Specifically modifying the RBPs could alter or extend the host range of phages otherwise exhibiting desired phenotypic properties. This study employed two different strategies to reprogram T7 phages ordinarily infecting commensal K12 *Escherichia coli* strains to infect pathogen-associated K1-capsule-expressing strains. The strategies were based on either plasmid-based homologous recombination or bacteriophage recombineering using electroporated DNA (BRED). Our work pursued the construction of two genetic designs: one replacing the *gp17* gene of T7, the other replacing *gp11*, *gp12*, and *gp17* of T7 with their K1F counterparts. Both strategies displayed successful integration of the K1F sequences into the T7 genome, detected by PCR screening. Multiple methods were utilised to select or enrich for chimeric phages incorporating the K1F *gp17* alone, including *trxA*, host-specificity, and CRISPR-Cas-based selection. Irrespective of the selection method, the above strategy yielded poorly reproducible phage propagation on the new host, indicating that the chimeric phage was less fit than the wild type and could not promote continual autonomous reproduction. Chimeric phages obtained from BRED incorporating *gp11-12* and *gp17*, however, all displayed infection in a 2-stage pattern, indicating the presence of both K1F and T7 phenotypes. This study shows that BRED can be used as a tool to quickly access the potential of new RBP constructs without the need to engineer sustainably replicating phages. Additionally, we show that solely repurposing the primary RBP is, in some cases, insufficient to produce a viable chimeric phage.

## 1. Introduction

The acceleration and spread of bacterial antibiotic resistance have enabled phage therapy, the use of bacteriophages (phages) as therapeutics for resistant bacterial infections, to develop and mature as a technology over the last two decades. Clinical trials are in progress in both the U.S. and Europe [1] and procedures to access compassionate use of phage therapy are already in place in many countries [2]. 

A potential hindrance to the large-scale use of phage therapy compared to traditional antibiotics is the high host specificity of phages. Traditional antibiotics can often target and treat entire classes of non-resistant bacteria, whereas phages are generally limited to a smaller subset of closely related strains [3]. Thus, phage therapy is currently highly individualised and requires identification of the pathogenic strain and subsequent selection of a suitable phage strain. However, advancements in the field of molecular biology are enabling researchers to edit genomes more efficiently than ever. Hence, there is a potential to design and engineer recombinant phages with new phenotypic properties, such as an altered or expanded host tropism.

A variety of methodologies have been developed for genetic engineering, many of which rely on the well-established principles of homologous recombination. Possible phage genome editing approaches include CRISPR-Cas, whole-genome synthesis, and genome assembly in yeast or in cell-free TX-TL systems [4]. 

The phage specificity is primarily determined by the receptor-binding proteins (RBPs) located on either tail fibres or tail spikes. These RBPs are the main focus of phage engineering, aiming to alter or expand the host tropism. A modular approach was employed by Ando et al. [5] to reprogram wild-type *Escherichia coli* (*E. coli*) phage T7 to infect different bacterial species, including *Yersinia* and *Klebsiella*. This approach included the synthesis of the entire phage genome followed by Gibson assembly and expression in yeast cells [5]. Demonstrating a different approach, Yosef et al. [6] extended the host range of phage T7, intended for DNA transduction and not replication, potentially targeting bacterial species that would not normally support the propagation of phage T7. Individual recombinant phage particles were engineered by homologous recombination, incorporating a specific RBP gene into their genome. The recombinant phages showed susceptibility to multiple *Klebsiella pneumoniae* and *Salmonella enterica* strains [6]. 

The primary goal of this work was to test multiple strategies to reprogram the host specificity of the bacteriophage T7. The ease and safety of propagating phage T7 on *E. coli* K12 allowed the accumulation of a vast amount of knowledge and the development of numerous techniques for this phage. This could make phage T7 an ideal candidate system to develop general phage traits (e.g., altered virulence or immunogenicity, or fusion with fluorescent labels or tags) for basic research or biotechnological purposes. Such a collection of altered T7 phages could execute specific phage-related tasks on other bacterial strains after quickly modifying host specificity. We report here the application of two different groups of strategies to reprogram the specificity of phage T7 for K1-capsule-expressing *E. coli* hosts. We found that if multiple phage genome segments needed to be replaced, linear-DNA mediated recombineering could yield the most promising results, even if the complete gene set required for full phage viability was unknown.

## 2. Materials and Methods

### 2.1. Strains, Buffers, and Media

Two *Escherichia coli* strains were used for phage propagation: *E. coli* K-12 MG1655 [7] and *E. coli* EV36, a K12/K1 hybrid developed by conjugation of the Hfr *kps*^+^ strain, which was kindly provided by Dr Eric R. Vimr [8]. Phage T7 was obtained from Professor Ian Molineux, and phage K1F was kindly provided by Dr Dean Scholl [9]. *E. coli* BW25113 ∆*trxA* were obtained from the Keio collection and were kindly donated (from Professor Alfonso Jaramillo’s lab). Bacteria were cultured in lysogeny broth (LB) [10]. Phage dilutions were made in buffer Φ80+, containing 0.1 M NaCl, 0.01 M Tris (pH7.9), 0.01 M CaCl_2_, and 0.01 M MgCl_2_ [11]. The TBE buffer contained 45 mM Tris, 45 mM boric acid, and 1 mM EDTA [10]. The TE buffer contained 10 mM Tris and 1 mM EDTA. Agar was used in a concentration of 1.5% in plates. SeaKem LE agarose (Lonza, Basel, Switzerland) used at 0.5% was applied as soft agarose overlay, always supplemented with 5 mM CaCl_2_ and 5 mM MgSO_4_. Antibiotics were used in the following end-concentrations: Ampicillin (Ap): 50 μg/mL, Chloramphenicol (Cm): 25 μg/mL, Kanamycin (Km): 25μg/mL. If not indicated otherwise, chemicals were obtained from Sigma-Aldrich (St. Louis, MO, USA).

### 2.2. Plasmids

For plasmid-mediated phage engineering, the donor DNA encoding a chimeric *gp17*, constructed by Aurelija Grigonyte [12], was further synthesised as a G-block by Integrated DNA Technologies (Leuven, Belgium) and cloned into pSB6A1 (iGEM http://parts.igem.org/Part:pSB6A1, accessed on 4 March 2021) using a Gibson assembly to make pSB6A1_T7-K1Fgp17. The G-block sequence is available in Supplementary Table S1.

Recombineering of linear fragments was mediated by λ-Red recombinases expressed from the pORTMAGE2 plasmid after heat-induction [13]. The plasmid pORTMAGE2 was a kind gift of Dr Ákos Nyeges.

Plasmids pCas9_T7gp17 and pCas9_T7gp17-2, used for counterselection against wild-type T7 phages, were constructed as described elsewhere [14]. Briefly, two complementary oligonucleotides (listed in Table S1) encoding the spacers with appropriate overhangs were phosphorylated, hybridised, and ligated into BsaI-digested pCas9 plasmids. The plasmid pCas9 was a kind gift from Prof. Luciano Marraffini (Addgene plasmid # 42876).

### 2.3. General Phage Protocols

Phages T7 and K1F were routinely propagated on *E. coli* strains MG1655 and EV36, respectively, using the protocol described earlier [15]. During all cases of phage growth, the liquid medium and soft agarose contained 5 mM CaCl_2_ and 5 mM MgSO_4_. For phage titering, a log10 serial dilution of the phage suspension was made and 10 μL of each dilution step was pipetted on top of solidified soft agarose, which contained the target bacterial strain at a 10^7^/mL initial concentration. Plaques were counted after incubating the plates (4 h for K1F, 16 h for T7) at 37 °C. For plaque PCR, plaques were aspirated with cut-off 200 µL pipette tips and resuspended in 100 µL of TBE buffer. After a 16 h incubation at 42 °C, 1 µL of the suspension was used in 10 µL PCR reactions to amplify various phage genome segments.

### 2.4. Phage DNA Preparation

Phage genome purification was carried out using a modification of earlier protocols [10,16]. First, a fresh T7 phage lysate was made from a 100 mL culture of *E. coli* MG1655 by infecting it with 4 × 10^5^ phages at OD_600_ = 0.5. After lysis, DNAase (Promega, Madison, WI, USA) and RNAaseA (Thermo Fisher Scientific, Waltham, MA, USA) were added at a final concentration of 2 µg/mL each and incubated for 2 h at 37 °C. Next, NaCl was added to a final concentration of 1 M and incubated for 2 h at 4 °C. Afterwards, the cell debris was removed by centrifugation at 11,000× *g* for 15 min at 4° C. PEG8000 was added to the supernatant at a final concentration of 10%, and the solution was incubated at 4 °C overnight. The precipitated phage particles were recovered by pelleting at 11,000× *g* for 15 min at 4 °C. The supernatant was discarded and the pellet was resuspended in 400 µL of TE. A quantity of 400 µL of buffered phenol was added, and the mixture was shaken for 10 min. After centrifugation at 10,000× *g* for 3 min, the aqueous (top) layer was recovered and an equal amount of phenol was added for two more rounds of phenol extraction. After phenol treatment, 300 µL of chloroform was added, shaken, and centrifuged for 1 min at 10,000× *g*. The aqueous (top) layer was recovered for another round of chloroform treatment. Next, 40 µL of 3 M sodium acetate was added to the recovered top phase, and phage DNA was precipitated with six volumes of 100% ethanol at room temperature. DNA was pelleted at 10,000× *g* for 12 min at room temperature. The supernatant was removed, and 1 mL of 70% ethanol was added and centrifuged for another 10 min. This step was repeated one more time. The pellet was dried and resuspended in 100 µL of TE buffer.

### 2.5. Plasmid-Mediated Phage Editing

Recombinant phages were obtained by two consecutive rounds of homologous recombination. Each round consisted of adding the phage at an MOI of <1 to a log phase bacterial culture at an OD600 of 0.3, previously transformed with the donor plasmid, and incubated at 37 °C with rotation (200 rpm). Following the bacterial clearance, the lysate was centrifuged at 3220× *g* for 15 min at 4 °C and subsequently passed through a 0.22 μm pore size filter.

Selection for recombinant T7/K1F chimeric phages was performed using the standard double overlay method with multiple approaches: marker-based selection on a thioredoxin-deficient bacterial host, *E. coli* BW25113 ∆*trxA*; selection on a K1-capsule-expressing host; and selection on a host expressing pCas9 targeting phage T7 wild-type tail fibres. Candidate plaques were picked and screened by PCR using primers AS072 and AS081 to confirm the presence of recombinant phages. The phages were recovered from the plaques into the TBE buffer (as described above), were re-grown on the same host in a liquid medium, and were re-plated in successive rounds with the intent of confirming the genotype and obtaining pure phage clones.

### 2.6. Phage Selection Using pCas9

The phage mix obtained from the plasmid-mediated phage editing was grown on *E. coli* EV36 harbouring either the pCas9_T7gp17 or the pCas9_T7gp17-2 plasmid. The obtained phage lysates were titered and plated on *E. coli* EV36 in appropriate dilutions to obtain individual plaques. Plaques were screened by PCR using primers T7tailFW and K1FtailRev to detect recombinant phages.

### 2.7. BRED

**Figure 1:**
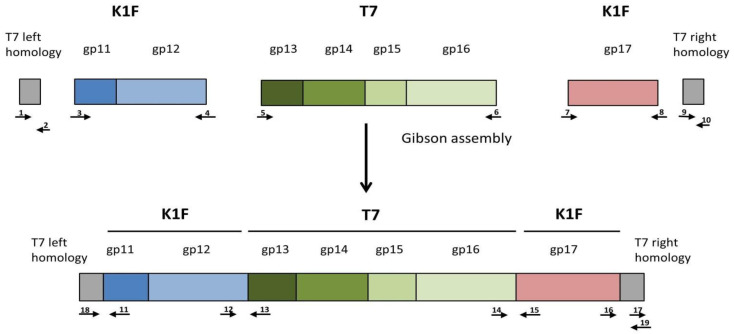
Construction of the genetic cassette designed to simultaneously alter *gp11*, *gp12*, and *gp17* of phage T7. The small arrows indicate the following primers used for cassette assembly: 1: T7gp10fw; 2: T7gp10rev; 3: K1Fgp11fw, 4: K1Fgp12rev; 5: T7gp13fw; 6: T7gp16rev; 7: K1Fgp17fw; 8: K1Fgp17rev; 9: T7gp18fw; 10: T7gp19revE. The following primers were used for joint verification: 11: K1Fgp11rev; 12: K1Fgp12fw; 13: T7gp13rev; 14: T7gp16fw; 15: K1Fgp17Rev(check); 16: K1fgp17fw(check); 17: T7gp19fw; 18: T7gp10fw(check); 19: T7gp19revE(check). Drawing not to scale.

Bacterial recombineering by electroporated DNA (BRED) [15] was used for the simultaneous modification of *gp11-12* and *gp17* genes of the T7 genome. The linear DNA used for this purpose was generated in three steps: first, PCR-amplification of the five segments shown in Figure 1; second, overlap-extension PCR-based fusion [17] of T7 left homology with *gp11-12* and *gp17* with T7 right homology; third, the NEBuilder (New England Biolabs, Ipswich, MA, USA) based assembly of three DNA fragments (T7 left homology + *gp11-12*; *gp13-16*; *gp17* + T7 right homology), each present at 0.18 pmol quantities (Figure 1). PCRs were carried out with Phusion DNA polymerase (Thermo Fisher Scientific, Waltham, MA, USA) at an annealing temperature of 58 °C. Primer sequences are listed in Table S1. The entire completed NEBuilder reaction was mixed with 50 µL (ca. 110 µg) of the purified T7 genomic DNA, and the mix was concentrated into 5 µL of TE by ethanol precipitation. For BRED, 2 µL of this concentrated mix was electroporated into thermally induced *E. coli* EV36/pORTMAGE2 electrocompetent cells. The electroporated cells were mixed with 3 mL of molten soft agarose, poured on LB + Ap plates, and incubated at 37 °C. The plaques that appeared were analysed by plaque-PCR using the following primer pairs to identify recombinants: gp10FWcheck + K1Fgp11Rev; K1Fgp12FW + T7gp13Rev; T7gp16FW + K1Fgp17Rev(check); and K1fgp17FW(check) + T7gp19Rev. The obtained agarose plugs were incubated at 42 °C overnight in 100 µL of TE for the diffusion of phages into the buffer. A quantity of 50 µL of this solution was added to 5 mL log phase culture of *E. coli* EV36pORTMAGE2 at 37 °C for phage propagation in an LB + Ap medium containing 5 mM CaCl_2_ and 5 mM MgSO_4_. After lysis, 1450 µL of the lysate was mixed with 50 µL of chloroform, pelleted, and the aqueous (top) layer was recovered and filtered sterile with a 0.22 μm pore size PVDF filter. For the next cycle of phage growth, 10 µL of this treated lysate was added to 5 mL of naive *E. coli* EV36pORTMAGE2 log phase culture, creating a 500× dilution.

In the alternative BRED protocol, 1 mL of electroporated culture from BRED was directly mixed with 5 mL of *E. coli* EV36 cells. After 2 h of shaking at 37 °C, the lysate was chloroform-treated and filtered sterile with a 0.22 μm pore size PVDF filter. For the next cycle of phage growth, either 5 or 500 µL of the lysate was mixed with *E. coli* EV36 corresponding to 1000× and 10× dilutions, respectively. Such growth cycles were repeated, as described in the Results.

## 3. Results

In this project, we tested multiple strategies to alter the host specificity of phage T7, to provide the capability of infecting K1-capsule-expressing *E. coli* cells. As a starting point, we analysed the growth and plaque-forming abilities of phage T7 and K1F on *E. coli* MG1655 and *E. coli* EV36 strains displaying the K12 and K1 capsules, respectively. The results, detailed in Table 1, indicated that the liquid-culture-mediated growth of T7 and K1F is only possible on *E. coli* MG1655 and EV36 strains, respectively. However, plaque formation in similar numbers was observed when switching the bacterial hosts (i.e., T7 on EV36 and K1F on MG1655), albeit yielding smaller plaque diameters. This indicated that the host specificities of the phages were not completely exclusive at the starting point of our experiments.

**Figure 2:**
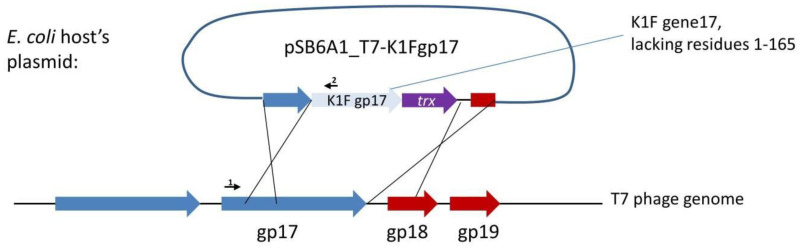
Strategy I, the genetic construct used for plasmid-mediated phage engineering. Large arrows depict genes. Small arrows represent the following primers: 1: AS072; 2: AS081.

In the case of phage T7, binding to the cell surface is ensured by the six tail fibres. Each tail fibre consists of three Gp17 proteins, all attached to the phage tail with their N-termini. The phage tail is made up of a dodecamer of the Gp11 and a hexamer of the Gp12 proteins. Gp7.3, an essential protein that is injected into the target cell, is also present in the tail in about 30 copies at an unknown location [18]. Our first strategy to completely switch the specificity of phage T7 to K1 hosts targeted the tail fibre using plasmid-mediated phage engineering, i.e., the growth of T7 on *E. coli* K12 cells harbouring a donor plasmid (pSB6A1_T7-K1Fgp17) that carries a modified *gp17* gene (Strategy I). This plasmid was designed to include the 860 C-terminal residues of the K1F *gp17* (possessing endosialidase activity), thioredoxin (*trxA*) for positive selection, and two flanking phage T7 homology regions (Figure 2). Sequence similarities between phage K1F and phage K1-5, another K1 capsule-targeting phage, showed that the 204 amino acids (AAs) of the N-terminal end of K1F Gp17 were not necessary for the correct folding of the enzymatic protein. We, therefore, designed homologies to obtain a fusion protein where the 165 AA N-terminal sequence of K1F *gp17* is replaced with the 162 AA N-terminal end of T7 *gp17*.

**Figure 3:**
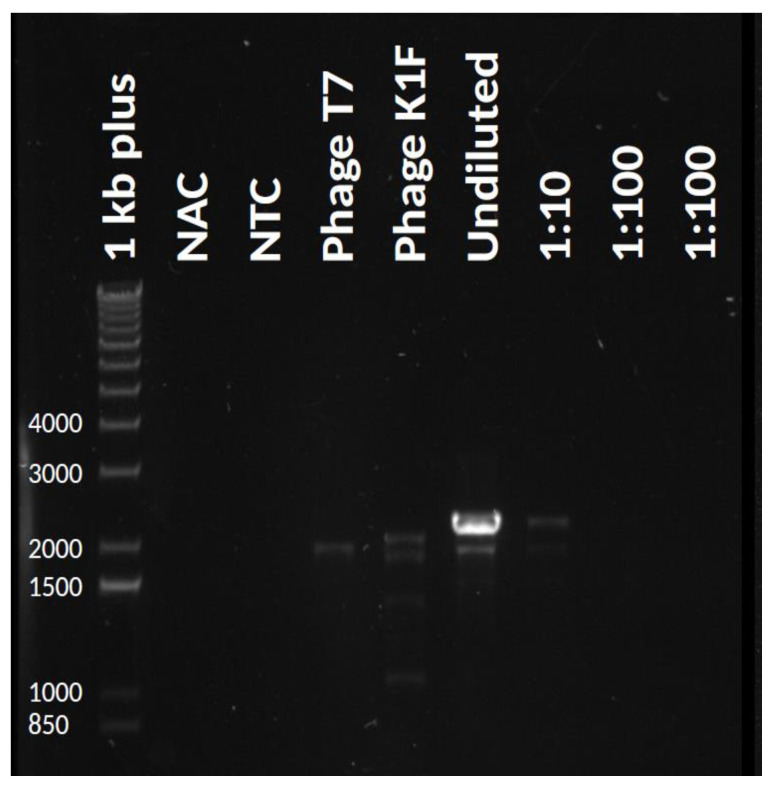
PCR analysis verifying the presence of the fusion *gp17* gene in the undiluted phage mix obtained by plasmid-mediated phage editing. An NAC (no amplification control) without taq polymerase and an NTC (no template control) without DNA template was included in each run. Marker: GeneRuler 1 kbp DNA Ladder Plus (Thermo Fisher Scientific, Waltham, MA, USA).

**Figure 4:**
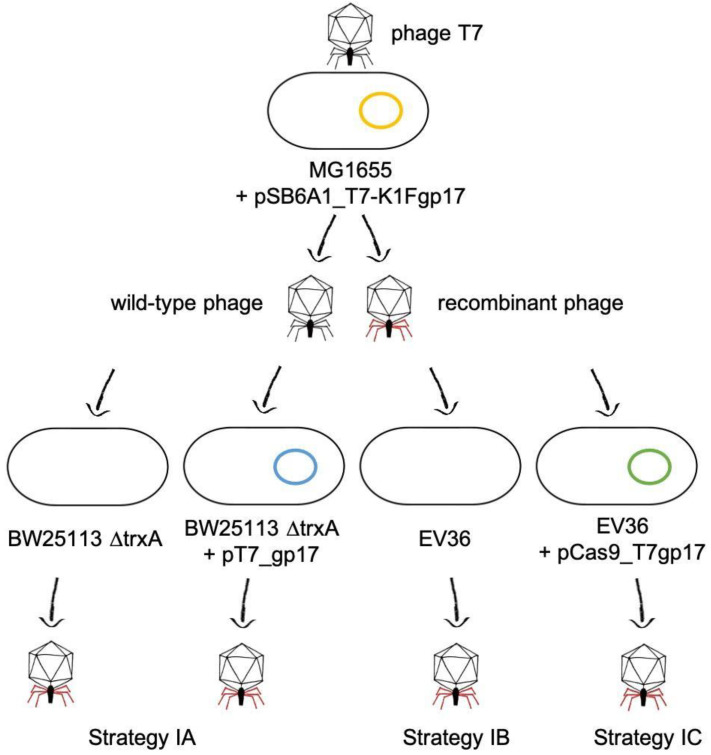
Plasmid-based homologous recombination and selection strategies used for phage editing.

Propagation of phage T7 on *E. coli* K-12 (MG1655)/pSB6A1_T7-K1Fgp17 yielded a phage lysate that contained detectable amounts of the fusion *gp17* gene, as verified by PCR using primers AS072 and AS081 (Figure 3). We applied three different strategies (IA-C) to select for or enrich the recombinants within the phage mix (Figure 4). Strategy IA consisted of phage growth on *E. coli* BW25113 ∆*trxA*, which lacks the thioredoxin gene. Since this gene is essential for phage replication, this host only allows the replication of phage genomes that have acquired a copy of the *trxA* gene. Unfortunately, PCR-detectability of the chimeric phage was lost over the first few rounds of phage propagation. The failure of this method can be, in part, attributed to the fact that our ∆*trxA* strain did not carry a K1 capsule and was, therefore, a suboptimal host for the propagation of the recombinant phage. Attempting to boost phage propagation by providing the Gp17 protein of T7 in trans did not solve this issue: the PCR signal also disappeared when growing the phages on *E. coli* BW25113 ∆*trxA* + pT7_gp17. 

Strategy IB was more straightforward; it relied on the potentially improved ability of the recombinant phage to lyse liquid cultures of *E. coli* EV36, a K1 capsule-expressing host. We propagated the phage mix on liquid cultures of *E. coli* EV36, measuring phage titers after every round of phage growth (even if no visible lysis occurred) using plated EV36. Overall, the phages displayed a weak and poorly reproducible ability to grow in liquid EV36 cultures. As apparent from Table 2, the titers measured in the course of three passages strongly fluctuated; furthermore, during the first and third steps, we only recovered the phages that we had mixed with the cells, indicating negligible phage propagation. A very similar titer pattern was observed when starting not with the complete phage lysate, but with a plaque obtained by plating the lysate on *E. coli* EV36. Importantly, in both series of propagations, the phage mix lost the PCR-positivity by the third passage. Overall, we concluded that propagating the phage mix on a K1-expressing host did not select for the recombinant phages and could not even maintain the fusion *gp17* genotype within the mix.

In Strategy IC, we intended to further increase the pressure enriching recombinant phages by applying CRISPR-Cas selection. We grew the phage mix on K1 capsule-expressing hosts that carried the *S. pneumoniae* CRISPR-Cas machinery targeted against either of two loci in the T7 *gp17* gene, not present in the fusion *gp17*. Growth of the phage mix on *E. coli* EV36/pCas9_T7gp17 or EV36/pCas9_T7gp17-2 was carried out both in liquid culture and on plates, as described in the Methods. In both cases, the PCR-positivity of the obtained phages was lost already after the first round of phage growth, irrespective of the guide RNA used.

At this point, it became evident that the recombinant phage was not viable, or at least its selective disadvantage compared to T7 was greater than the selective pressure exerted by any of the three systems described above. A potential explanation and solution were provided by the work of Ando et al. [5], who made similar observations when replacing the *gp17* gene of T7 with that of *Klebsiella* phage K11. In their work, the recombinant phages were not viable unless *gp11* and *gp12* (the genes encoding the adaptor and the nozzle proteins of the phage tail, respectively) were also replaced by their K11 counterparts. The authors explained this requirement by the fact that the *gp17*-encoded tail fibres attach to the interface between the Gp11 and Gp12 proteins. In light of this information, we redesigned the donor plasmid to comprise the K1F derived *gp11*, *gp12*, and *gp17* genes, as well as the T7-derived *gp13, gp14, gp15*, and *gp16* genes, as shown in Figure 1. The gene set was to be flanked by two homologous ends allowing its entry into the T7 genome to replace the native *gp11*-*gp17* segment.

**Figure 5:**
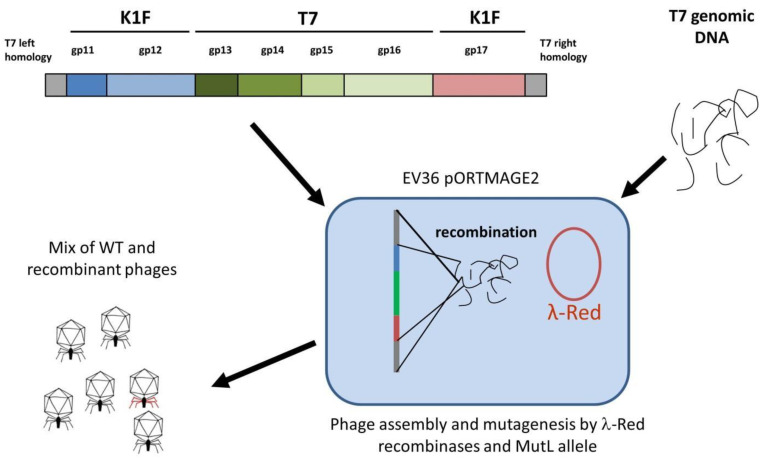
Strategy II, relying on bacteriophage recombineering using electroporated DNA. The linear DNA cassette comprising the planned genetic changes (**upper left**), is mixed with phage genomic DNA (**upper right**), and is electroporated into *E. coli* EV36 cells expressing the λ-Red recombinases from the pORTMAGE2 plasmid (**centre**). The recombinase assembles both WT genomes (yielding WT phage particles, **lower left**) and genomes incorporating the linear DNA cassette (yielding recombinant phages, **lower left**, marked with red tail fibres).

The donor plasmid was to be constructed as a four-way assembly (T7 left homology + *gp11*-*12*; *gp13-16*; *gp17* + T7 right homology; bacterial artificial chromosome segment) (Figure S1) using the method of Gibson et al. [19]. Gel electrophoresis of the reaction product and PCR of the assembly joints indicated that the 14.2 kbp-long linear cassette was most likely assembled (Figure S2). However, cloning it into pBeloBAC11 was not successful, irrespective of whether *E. coli* MDS42, MG1655, or EV36 was used as a host, possibly due to the toxicity of the construct. We, therefore, abandoned plasmid-mediated phage editing and switched to Strategy II (Figure 5), which relies on bacteriophage recombineering using electroporated DNA (BRED) [20]. This strategy builds on the fact that if purified phage DNA is electroporated into an *E. coli* cell expressing the λ-Red recombinases, complete phage genomes are assembled, yielding phage plaques within the bacterial lawn. If a linear DNA fragment with appropriate homology arms is included in the electroporation mix, a certain fraction of the obtained plaques will contain recombinant phages, detectable by plaque-PCR.

**Figure 6:**
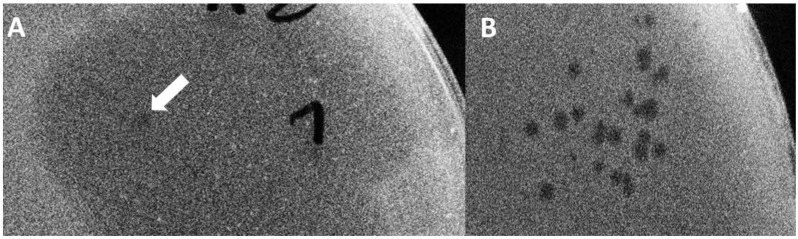
(**A**) A plaque of the recombinant phage displaying the two-stage phenotype on an *E. coli* EV36 host. The arrow marks the clear centre. (**B**) Plaques of phage T7 on *E. coli* EV36.

For strategy IIA, *E. coli* EV36/pORTMAGE2 cells were electroporated with a mix of T7 genomic DNA and the linear DNA fragment, as shown in Figure 5. After plating and overnight growth, a total of 14 large (>10 mm) clear plaques were observable. We analysed all plaques by four distinct PCR reactions (listed in Materials and Methods), screening for the presence of the four T7/K1F joints. We found that all of the plaques contained at least one of the two transgenic segments (i.e., *gp11-12* or *gp17*) and four of them contained both integrated into the T7 genome at the correct locus (Figure S3). Interestingly, small plaques formed by wild-type T7 did not emerge (otherwise seen when the phage genome is transformed without linear DNA). A double-positive plaque was picked, and the phages were recovered and further propagated on liquid *E. coli* EV36 culture to verify its growth ability and to enrich for virions containing the altered tail fibre. No lysis was observable in the liquid medium, and the obtained plaques displayed the absence of *gp17* and *gp11-12* genes, as analysed by PCR. As an alternative (Strategy IIB), the phages recovered from the plaque were grown on *E. coli* EV36/pORTMAGE2 to provide further possibilities of recombination (by the λ-Red recombinases) and an increased rate of point mutations (by the *mutL* allele). Growth on liquid *E. coli* EV36/pORTMAGE2 culture was possible, as the cells were consistently lysed. However, the transgenic segments of *gp17* and *gp11-12* were lost in the first and second round of phage growth, respectively. Genes of the T7 phage were readily detectable by PCR in all rounds of phage growth (Figure S4). The continued lysis of the non-corresponding host by phage T7 is explained by the presence of λ-Red recombinases. The expression of λ-Red in bacterial cells has recently been shown to provide sufficient genetic reorganisation to lyse non-canonical hosts [21] (in a control experiment, we found that serial propagation of T7 on *E. coli* EV36/pORTMAGE2 liquid cultures led to full lysis starting from the second round, without any linear DNA fragment added; data not shown). The obtained phages nevertheless retained their ability to lyse *E. coli* MG1655, as well. The activity of the K1F*gp17* was still present after the end of the serial phage growth experiment in the form of two-stage plaques (Table 3, Figure 6), assuming some lasting genetic change did occur. The same could not be seen when wild-type T7 was assembled without K1F gene fragments.

Finally, in Strategy IIC, we repeated the BRED, but instead of plating the transformed bacterial mix and picking a plaque, we grew the bacteria (and the emerging phages) directly in a liquid culture. This avoids the bottleneck introduced by randomly picking a plaque and allows the competition of all putative recombinant and wild-type T7 phages within a single mix. Growth took place without antibiotic selection to promote the quick loss of the pORTMAGE2 plasmid. In this case, although bacterial lysis was not always visible in the successive rounds of growth, phages were always detectable, despite the lack of pORTMAGE2. However, transgenic fragments were quickly lost from the phage genome. In all versions of Strategy II, the obtained plaques displayed a two-stage phenotype, usually starting from the second or third round of growth. This meant a small, clear centre surrounded by a turbid halo assuming the endosialidase activity of the K1F Gp17 protein [22] and the area of both kept increasing day-by-day (Figure 6). The observations made with BRED experiments are summarised in Table 3.

## 4. Discussion

Ever since the discovery of bacteriophages, knowledge concerning their host range has been vital for biomedical applications for at least two reasons. On the one hand, phage typing has long been used in microbial diagnostics to identify and classify the pathogens isolated from bacterial infections [23]. On the other hand, phage therapy requires the administration of phages capable of lysing the targeted pathogen. These needs explain the increased attention paid towards phages that display a broad host range [24] or a host range dependent on environmental factors, like temperature [25]. Later, spontaneously arising host range mutants were often described [26,27], and with the advent of DNA sequencing, the mutations responsible for the changes were also successfully identified [28,29]. With the accumulation of sequencing data, comparative genomic studies of phage sequences became possible. These revealed that in certain cases, exchanges of large genomic segments are responsible for host switching [30]. Such information, along with the development of phage engineering techniques, allowed the reprogramming of the host range of certain phages by replacing host-range-determinant DNA segments [5,31]. Recently, the structure-guided design of receptor-binding proteins has proven to offer an even more advanced method of redirecting phages towards new hosts [32].

Our work fits into this series by providing an example of transferring one or more large genomic segments between phages to alter the host tropism of the recipient phage. In the course of this project, we tested two groups of strategies and two different genetic constructs. Strategy I applied plasmid-based phage editing using either *trxA* (IA), host-specificity (IB), or CRISPR-Cas-based selection (IC). Strategy II relied on BRED, using a host-specificity-based selection of the recombinant phage. For Strategies IIA and IIB, a single positive plaque was picked for the outgrowth of the putative recombinants on cells either lacking or containing the λ-Red recombinase enzymes, respectively. Alternatively, outgrowth of the entire phage lysate was attempted in the absence of λ-Red recombinase enzymes for Strategy IIC. 

To the best of our knowledge, this is the first published work aiming to tune phage T7 tropism towards a K1-capsule-expressing host. This type of specificity-change could bear clinical relevance in the future, when the rapid redirection of certain well-established phages towards novel bacterial pathogens is required. From the molecular genetic point of view, all six strategies accomplished the goal of producing detectable recombinant constructs. Since well-established techniques were used for their selection, we attribute the differences seen in the sustainability of the constructs not to differences of the selection methods themselves but instead to the differences in the genetic design. Strategy I only altered *gp17* and left the 5′ end of the gene unchanged, while Strategy II applied the complete exchange of the *gp17* gene, along with that of *gp11* and *gp12*. Since the latter strategy yielded plaques that stood closest to the phenotype of completely lysing the new host, we conclude that modification of either *gp11* or *gp12,* or both, are required, in addition to that of *gp17* for the reprogramming of T7 to lyse K1-type hosts. The temporary nature of such lysis, however, indicates that these genetic changes were still not sufficient to develop a stable viable phage. Although this may be a shortcoming in some instances, it is satisfactory for numerous experimental setups. The most prominent example is the engineering of transducing phages or phage libraries, where efficient phage binding, DNA injection, and the overcoming of defence mechanisms without autonomous phage replication are enough to meet the requirements of practical applications [6]. 

Besides yielding unstable reprogrammed phages, our work also provides findings that may help design future host switching experiments. First, we observed that the presence of pORTMAGE is capable of allowing the growth of T7 on *E. coli* EV36 in liquid, irrespective of donor DNA. This is in line with recent observations reporting that λ-Red recombinase expression enables T7-like bacteriophages that do not normally propagate in *E. coli* to be recovered following genome transfection [21]. Including donor DNA in the transformation mix nevertheless exerted the temporary effect of yielding large clear plaques, which was eventually lost. This means that (a) the genes responsible for efficient lysis of *E. coli* EV36 are present; and (b) no pure, viable recombinant phage, which could pass on its genetic composition to its offspring, was made. A mixed infection (T7 + recombinant phages) or the integration of donor DNA into the bacterial host genome probably provided a genetic background that resulted in the formation of the large clear plaques seen in the first plating of Strategy IIA. Second, the observed two-stage plaques indicated the expression of the K1F *gp17* gene [22]. According to our hypothesis, *gp17* was transiently expressed, and Gp17 enzymatically broke down the capsule, forming the turbid halo. This allowed the access of T7 to the cell surface, which formed the clear centre of the two-stage plaques. However, the gene construct is unstable; the proteins encoded by the construct cannot assemble to yield viable phage particles alone. If a helper T7 is present, it can package the construct and transfer, but again, it will not form viable particles alone. Therefore, recombinant constructs are quickly lost from the phage mix. If the infected strain harbours λ-Red recombinases, it can protect the transformed *gp17* gene cassette and potentially integrate *gp17* transferred by the helper T7 phage into the bacterial genome. This allows transient production of endosialidase, which, upon cell lysis, will break down the capsule of neighbouring cells. The case of a mixed infection (T7 + recombinant phages) is supported by the fact that even when propagating phages derived from isolated plaques declared recombinant by PCR, wt T7 phages eventually become dominant and even exclusive after a few cycles of growth on EV36. BRED is known to produce mixed plaques [20], since both WT and recombinant phage genomes can be assembled in the same transformed host, which warrants that wt T7 is available as a helper phage during the initial growth of the recombinants. Transferring to liquid phase, however, lowers the chances of co-infection due to the effect of dilution, leading to the eventual cessation of the packaging of the recombinant phage genome into T7-virions.

Finally, although a stable phage displaying host switching was not assembled in this work, the large clear plaques are a strong indication that the necessary genes required for serial lysis of a non-canonical host were all present. The T7 engineered phage, even though it was not stable for the long term, still presented broader tropism and could efficiently target EV36 presenting the K1 capsule, which is normally a host for the K1F phage. The use of BRED for this purpose could be a quick way to test the potential use of a new tail fibre without the need to engineer a stable phage.

Concerning the future, the information collected to this point could provide a basis for the further enhancement of the hybrid construct, ultimately leading to a stable T7-K1F chimeric phage. The two most straightforward explanations of the instability of our chimera could be either the inadequate expression of genes present on the construct or the inappropriate assembly of the encoded proteins due to their potentially incompatible adjacent surfaces. Although we cannot completely exclude the former, earlier reports on reorganizing genes within a phage genome indicated a relatively high level of robustness [33,34]. Perhaps more likely is the incompatibility of neighbouring protein surfaces. This would suggest that modular phage reorganization, which works quite effectively in certain cases [5], is insufficient in others and demands sub-modular tuning for successful host-switching. The accumulation of protein structural data and the development of novel combinatorial genome editing techniques will certainly aid both the design-based and the selection-based strategies aiming to solve such issues in the future. 

## Figures and Tables

**Table 1:** Growth properties of phages T7 and K1F.

T7	MG1665	EV36	K1F	MG1665	EV36
canonical host	yes	no	canonical host	no	yes
able to lyse	yes	yes(!)	able to lyse	yes(!)	yes
plaque size *	large	small	plaque size *	small	large
able to clear liquid culture	yes	no	able to clear liquid culture	no	yes

* Small plaques: 1–2 mm, large plaques: 13–15 mm in diameter after 12–16 h of incubation.

**Table 2:** Phage titers obtained by growing the recombinant phage mix (obtained via the recombination step shown on the top of Figure 4) on *E. coli* EV36.

	Titer	Titer after First Passage (I)	Titer after Second Passage (II)	Titer after Third Passage (III)
original K1F/T7 lysate (o)	1.6 × 10^10^	8 × 10^4^	6 × 10^6^	4× 10^4^
plaque derived K1F/T7 (1)	---------	2 × 10^4^	6 × 10^6^	1 × 10^4^

**Table 3:** Summary of liquid bacterial lysis experiments with phages obtained by BRED.

	Isolated from Plaque; Grown without pORTMAGE	Isolated from Plaque; Grown with pORTMAGE	Directly from Electroporated Culture; Grown without pORTMAGE
	(Strategy IIA)	(Strategy IIB)	(Strategy IIC)
sustainable ^1^	no	yes	yes
sustainable on pORTMAGE-free cells	no	no	yes
clearing liquid culture	no	yes	inconsistent ^2^
two-stage plaques	yes	yes	yes
T7 tail genes present	yes	yes	yes
K1F tail genes	lost	lost	lost

^1^ Sustainability: capability of maintaining ≥10^6^/mL phage titers during eight rounds of propagation on the indicated host. ^2^ Inconsistency: phage propagation does not always result in the noticeable decrease of bacterial density.

## Data Availability

All the data are available upon request.

## Supplementary Materials

The following are available online at https://www.mdpi.com/article/10.3390/biology10060556/s1: Table S1. Primers used in this study, Figure S1. Gel electrophoresis of PCR fragments generated to construct the pBeloBAC11-based donor plasmid, Figure S2. Verification of donor fragment assembly, Figure S3. PCR-validation of the genetic fusion between K1F and T7 genes, Figure S4. PCR-validation of the T7 gp17 gene present in the phage lysate, Uncut gel photos list in the Supplementary Materials.

## References

[1] McCallin S., Sacher J.C., Zheng J., Chan B.K. (2019). Current State of Compassionate Phage Therapy. Viruses.

[2] Fauconnier A. (2019). Phage Therapy Regulation: From Night to Dawn. Viruses.

[3] Nóbrega F., Vlot M., de Jonge P.A., Dreesens L.L., Beaumont H.J.E., Lavigne R., Dutilh B.E., Brouns S.J.J. (2018). Targeting mechanisms of tailed bacteriophages. Nat. Rev. Genet..

[4] Pires D.P., Cleto S., Sillankorva S., Azeredo J., Lu T.K. (2016). Genetically Engineered Phages: A Review of Advances over the Last Decade. Microbiol. Mol. Biol. Rev..

[5] Ando H., Lemire S., Pires D.P., Lu T.K. (2015). Engineering Modular Viral Scaffolds for Targeted Bacterial Population Editing. Cell Syst..

[6] Yosef I., Goren M.G., Globus R., Molshanski-Mor S., Qimron U. (2017). Extending the Host Range of Bacteriophage Particles for DNA Transduction. Mol. Cell.

[7] Blattner F.R., Plunkett G., Bloch C.A., Perna N.T., Burland V., Riley M., Collado-Vides J., Glasner J.D., Rode C.K., Mayhew G.F. (1997). The Complete Genome Sequence of Escherichia coli K-12. Science.

[8] Vimr E.R., A Troy F. (1985). Regulation of sialic acid metabolism in Escherichia coli: Role of N-acylneuraminate pyruvate-lyase. J. Bacteriol..

[9] Scholl D., Merril C. (2005). The Genome of Bacteriophage K1F, a T7-Like Phage That Has Acquired the Ability to Replicate on K1 Strains of Escherichia coli. J. Bacteriol..

[10] Sambrook J., Fritch E.F., Maniatis T. (1987). Molecular Cloning. A Laboratory Manual.

[11] Blattner F., Fiandt M., Hass K., Twose P., Szybalski W. (1974). Deletions and insertions in the immunity region of coliphage lambda: Revised measurement of the promoter-startpoint distance. Virology.

[12] Grigonyte A.M., Harrison C., MacDonald P.R., Montero-Blay A., Tridgett M., Duncan J., Sagona A.P., Constantinidou C., Jaramillo A., Millard A. (2020). Comparison of CRISPR and Marker-Based Methods for the Engineering of Phage T7. Viruses.

[13] Nyerges Ákos, Csörgő B., Nagy I., Bálint B., Bihari P., Lázár V., Apjok G., Umenhoffer K., Bogos B., Pósfai G. (2016). A highly precise and portable genome engineering method allows comparison of mutational effects across bacterial species. Proc. Natl. Acad. Sci. USA.

[14] Jiang W., Bikard D., Cox D., Zhang F., Marraffini L.A. (2013). RNA-guided editing of bacterial genomes using CRISPR-Cas systems. Nat. Biotechnol..

[15] Fehér T., Karcagi I., Blattner F.R., Pósfai G. (2012). Bacteriophage recombineering in the lytic state using the lambda red recombinases. Microb. Biotechnol..

[16] Lech K., Ausubel F.M., Brent R., Kingston R.E., Moore D.D., Seidman J.G., Smith J.A., Struhl K. (1987). Making Phage DNA from Liquid Lysates. Current Protocols in Molecular Biology.

[17] Horton R.M., Hunt H.D., Ho S.N., Pullen J.K., Pease L.R. (1989). Engineering hybrid genes without the use of restriction enzymes: Gene splicing by overlap extension. Gene.

[18] Molineux I.J., Calendar R. (2006). The T7 group. The Bacteriophages.

[19] Gibson D.G., Young L., Chuang R.Y., Venter J.C., Hutchison C.A., Smith H.O. (2009). Enzymatic assembly of DNA molecules up to several hundred kilobases. Nat. Methods.

[20] Marinelli L.J., Piuri M., Swigoňová Z., Balachandran A., Oldfield L.M., van Kessel J., Hatfull G.F. (2008). BRED: A Simple and Powerful Tool for Constructing Mutant and Recombinant Bacteriophage Genomes. PLoS ONE.

[21] Jensen J.D., Parks A.R., Adhya S., Rattray A.J. (2020). λ Recombineering Used to Engineer the Genome of Phage T7. Antibiotics.

[22] Bessler W., Fehmel F., Freund-Mölbert E., Knüfermann H., Stirm S. (1975). Escherichia coli capsule bacteriophages. IV. Free capsule depolymerase 29. J. Virol..

[23] Chirakadze I., Perets A., Ahmed R. (2009). Phage Typing. Methods Mol. Biol..

[24] Lazarus A.S., Gunnison J.B. (1947). The action of Pasteurella pestis bacteriophage on Pasteurella, Salmonella, and Shigella. J. Bacteriol..

[25] Gunnison J.B., Larson A., Lazarus A.S. (1951). Rapid Differentiation Between Pasteurella Pestis and Pasteurella Pseudotuberculosis by Action of Bacteriophage. J. Infect. Dis..

[26] Iida S. (1984). Bacteriophage P1 carries two related sets of genes determining its host range in the invertible c segment of its genome. Virology.

[27] Morona R., Henning U. (1984). Host range mutants of bacteriophage Ox2 can use two different outer membrane proteins of Escherichia coli K-12 as receptors. J. Bacteriol..

[28] Tétart F., Repoila F., Monod C., Krisch H. (1996). Bacteriophage T4 Host Range is Expanded by Duplications of a Small Domain of the Tail Fiber Adhesin. J. Mol. Biol..

[29] Werts C., Michel V., Hofnung M., Charbit A. (1994). Adsorption of bacteriophage lambda on the LamB protein of Escherichia coli K-12: Point mutations in gene J of lambda responsible for extended host range. J. Bacteriol..

[30] Garcia E., Elliott J.M., Ramanculov E., Chain P.S., Chu M.C., Molineux I.J. (2003). The genome sequence of Yersinia pestis bacteriophage phiA1122 reveals an intimate history with the coliphage T3 and T7 genomes. J. Bacteriol..

[31] Chen M., Zhang L., Abdelgader S.A., Yu L., Xu J., Yao H., Lu C., Zhang W. (2017). Alterations in gp37 Expand the Host Range of a T4-Like Phage. Appl. Environ. Microbiol..

[32] Dunne M., Rupf B., Tala M., Qabrati X., Ernst P., Shen Y., Sumrall E., Heeb L., Plückthun A., Loessner M.J. (2019). Reprogramming Bacteriophage Host Range through Structure-Guided Design of Chimeric Receptor Binding Proteins. Cell Rep..

[33] Chan L.Y., Kosuri S., Endy D. (2005). Refactoring bacteriophage T7. Mol. Syst. Biol..

[34] Endy D., You L., Yin J., Molineux I.J. (2000). Computation, prediction, and experimental tests of fitness for bacteriophage T7 mutants with permuted genomes. Proc. Natl. Acad. Sci. USA.

