# Supplementary material for: Analysing Parallel Strategies to Alter the Host Specificity of Bacteriophage T7
Source: Biology (Basel). 2021 Jun 20;10(6):556. doi: 10.3390/biology10060556 (PMC8234382; doi:10.3390/biology10060556)
Supplement: Supplementary file 1 [file biology-10-00556-s001.zip › Supplementary Material_v2.pdf]

Supplementary Material

Supplementary Figures

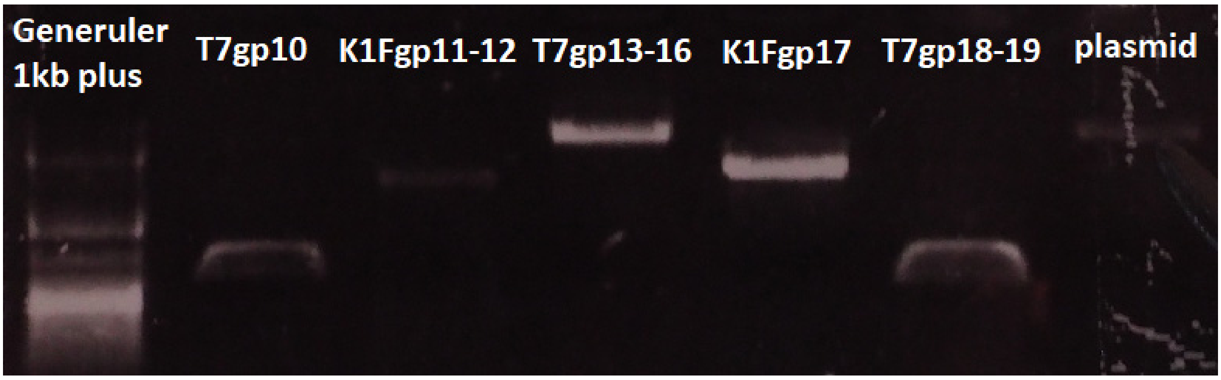

Figure S1. Gel electrophoresis of PCR fragments generated to construct the pBeloBAC11-based donor plasmid harbouring K1Fgp11-12, T7gp13-16 and K1Fgp17, flanked by two T7 genomic segments (gp10 and gp18-19) serving as homologies. Marker: GeneRuler 1 kbp DNA Ladder Plus (Thermo Fisher Scientific, Waltham, MA, USA).

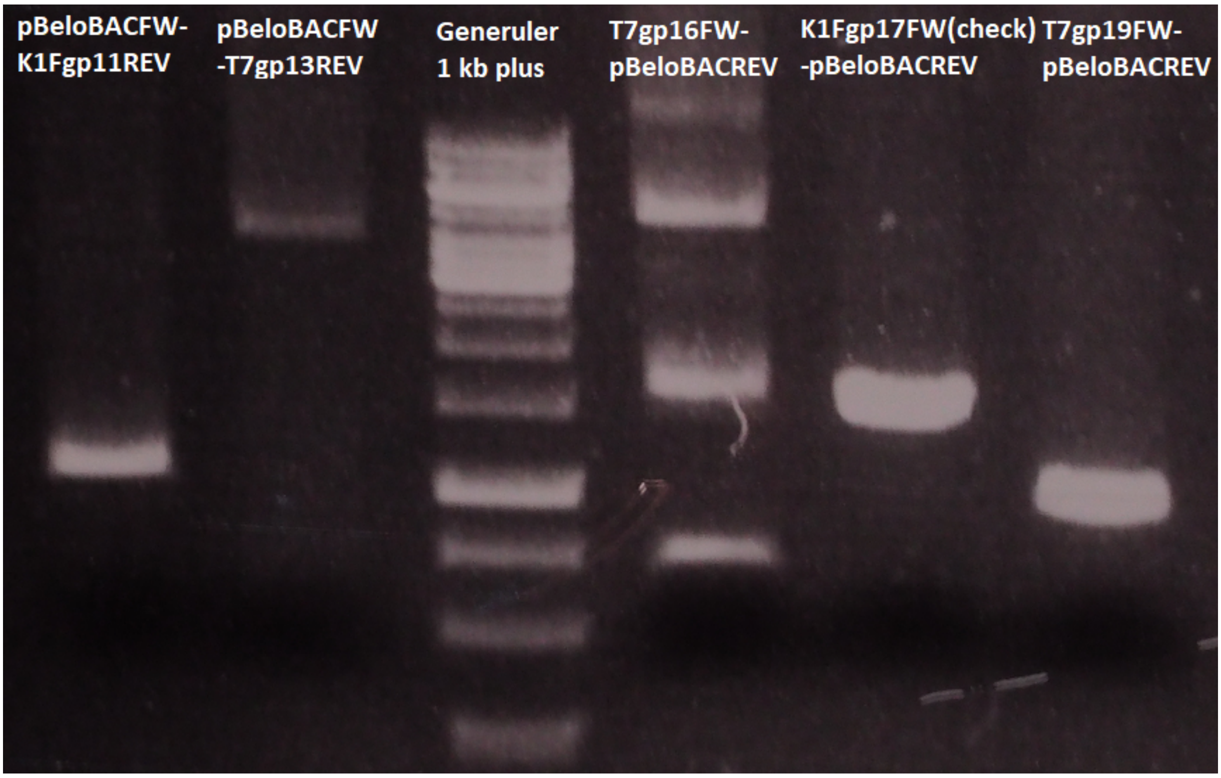

Figure S2. Verification of donor fragment assembly. Gel electrophoresis of the PCR fragments generated by amplifying the joints of the completed DNA fragment, obtained by overlap-extension PCR-based fusion and NEBuilder assembly. Marker: GeneRuler 1 kbp DNA Ladder Plus (Thermo Fisher Scientific, Waltham, MA, USA).

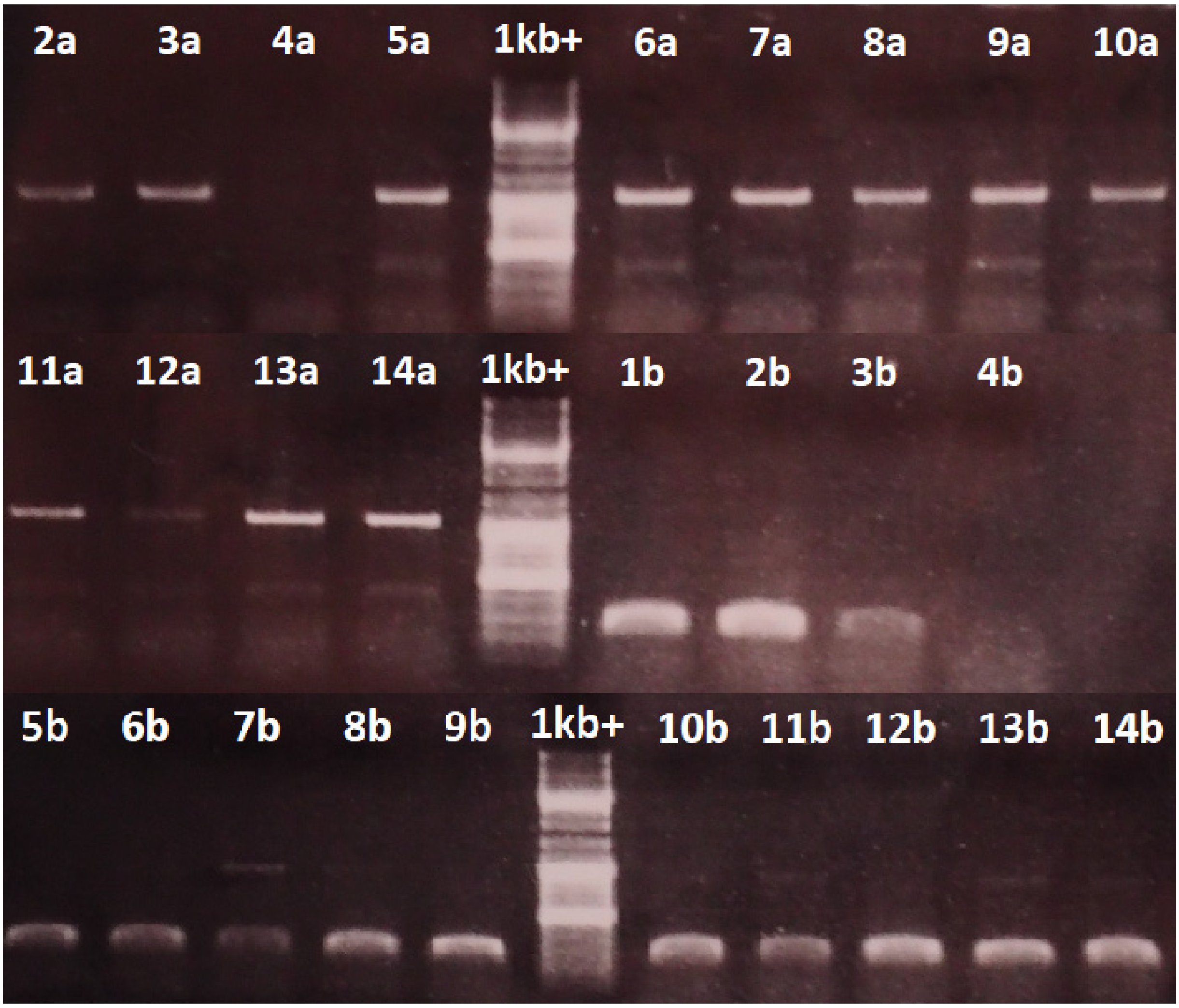

Figure S3. PCR-validation of the genetic fusion between K1F and T7 genes. Gel electrophoresis of the products of plaque PCRs is shown. Numbers 2a-14a: plaque-PCRs made with primers T7gp10FW(check) and K1Fgp11REV. Numbers 1b to 14b: plaque-PCRs of the same plaques made with primers K1Fgp17FW(check) and T7gp19REV(check). Note that plaque no. 7 gave relatively strong bands with both primer pairs. Marker: GeneRuler 1 kbp DNA Ladder Plus (Thermo Fisher Scientific, Waltham, MA, USA).

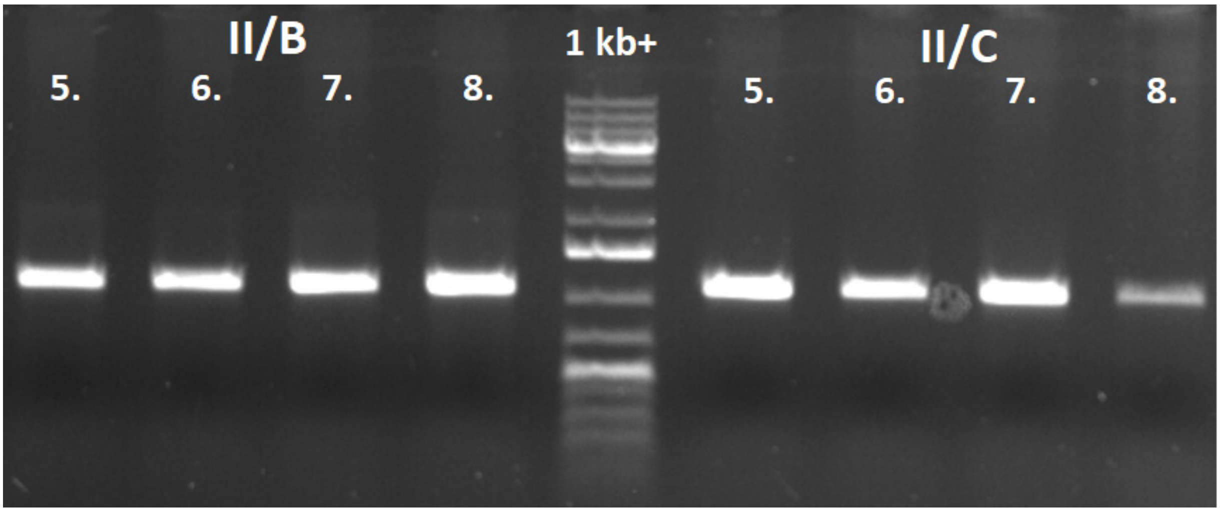

Figure S4.PCR-validation of the T7 gp17 gene present in the phage lysate. Gel electrophoresis of the products of plaque PCRs made with 50x diluted phage lysates using primers T7tailFW and T7tailREV. Numbers 5-8 indicate the rounds of phage growth after which the sampling was made, numbers II/B and II/C indicate the two experimental strategies. Marker: GeneRuler 1 kbp DNA Ladder Plus (Thermo Fisher Scientific, Waltham, MA, USA).

Uncut gel photos:

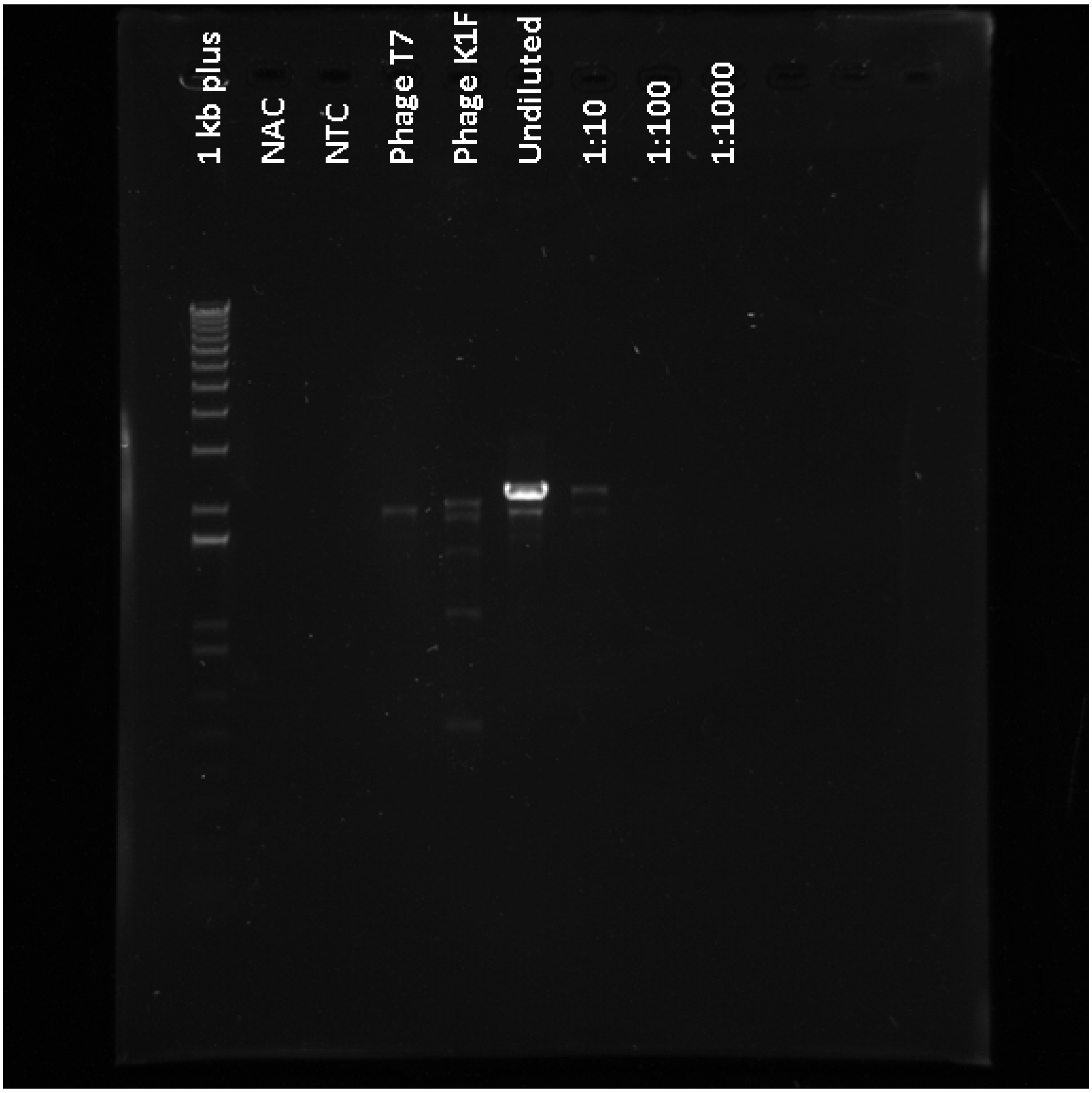

Figure 2 uncut.

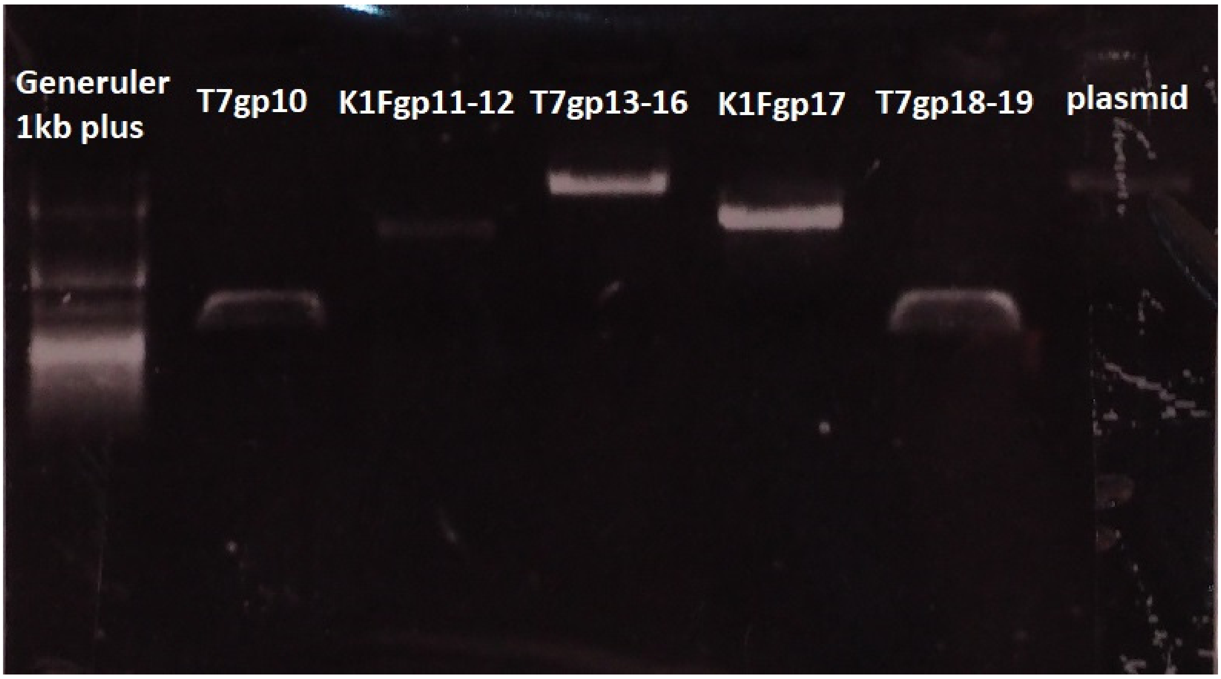

Figure S1 uncut.

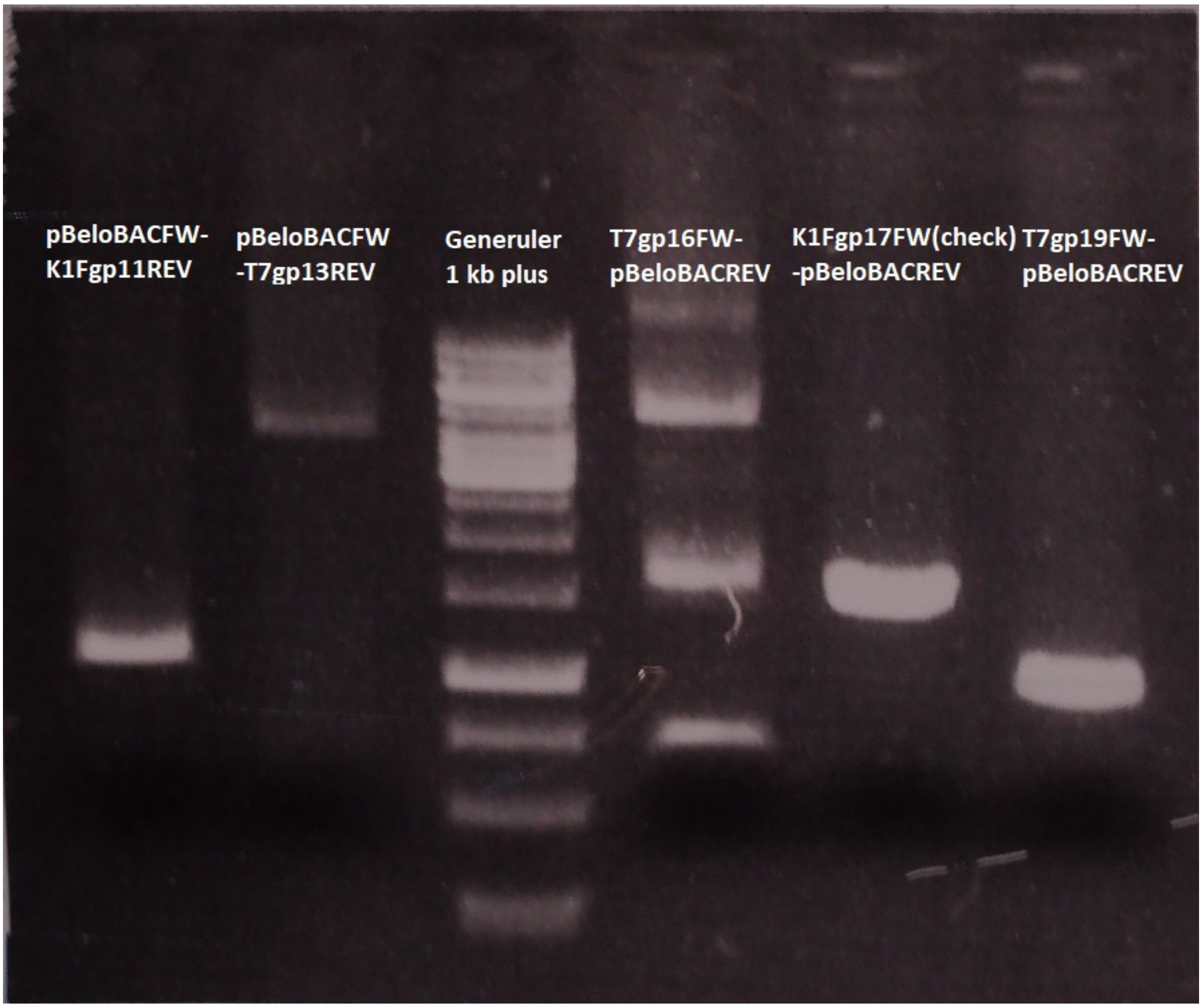

Figure S2 uncut.

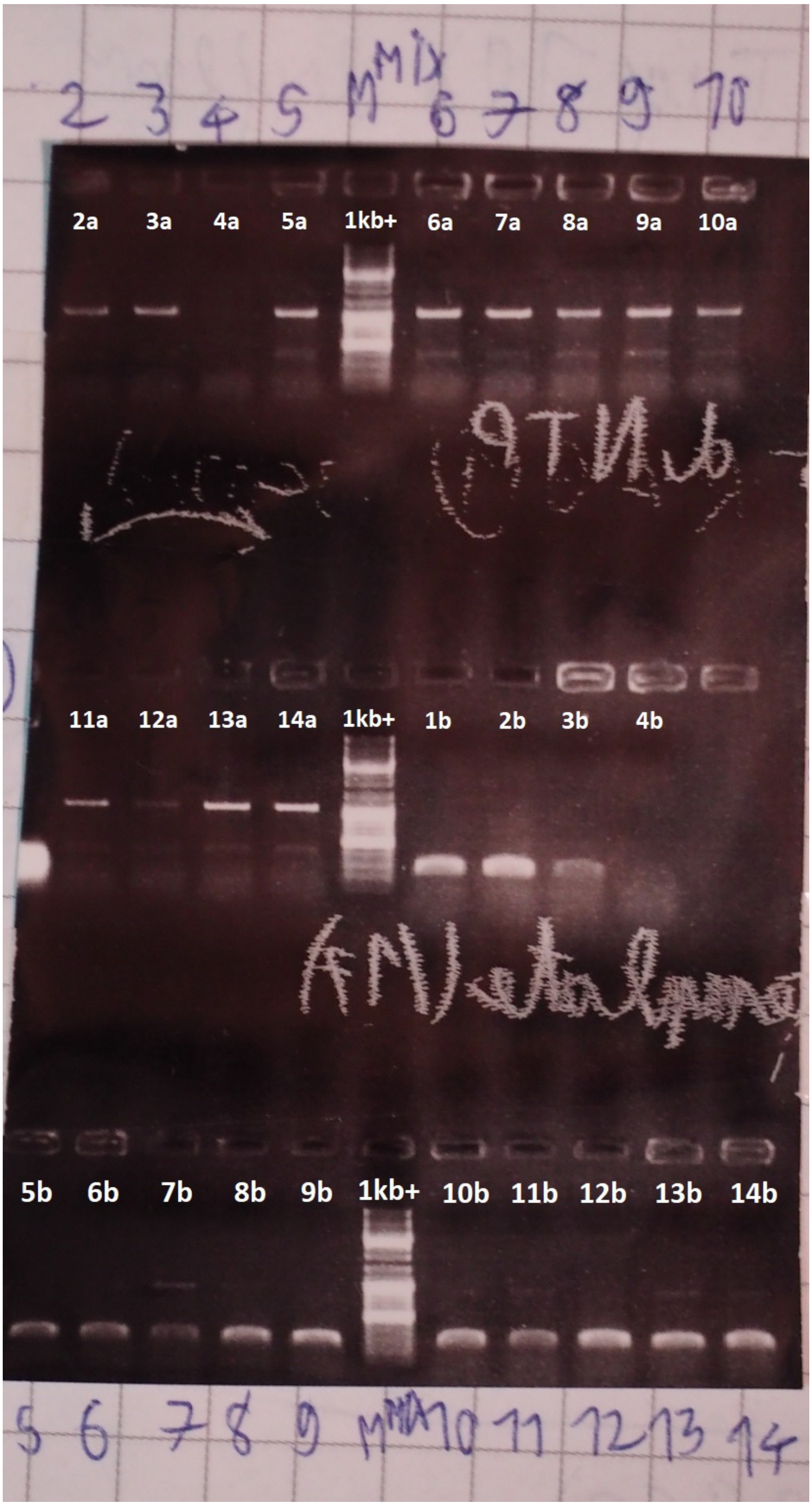

Figure S3 uncut.

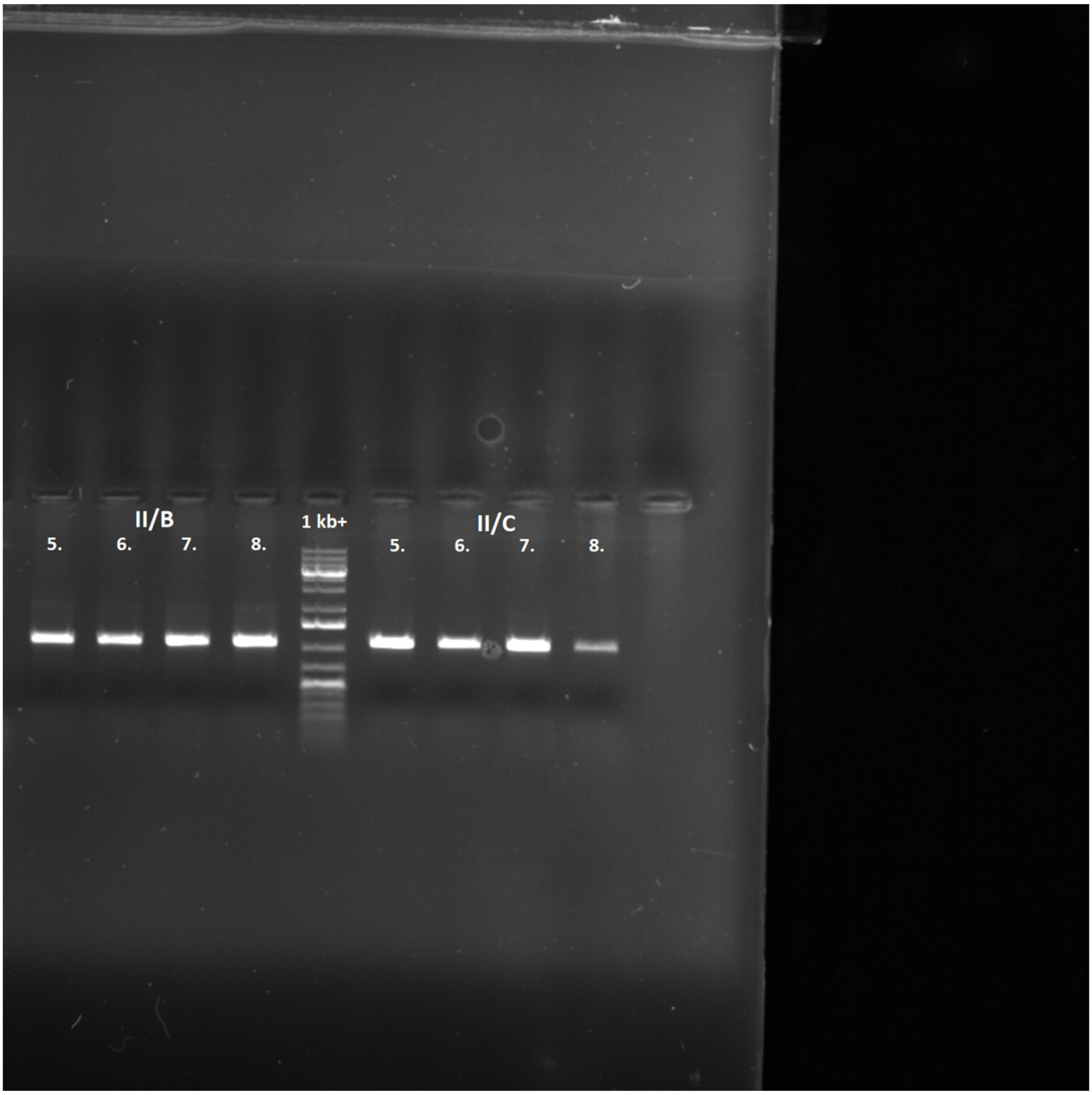

Figure S4 uncut.
